# ATF3 promotes erastin-induced ferroptosis by suppressing system Xc^–^

**DOI:** 10.1038/s41418-019-0380-z

**Published:** 2019-07-04

**Authors:** Liyuan Wang, Yichen Liu, Tingting Du, Heng Yang, Lei Lei, Mengqi Guo, Han-Fei Ding, Junran Zhang, Hongbo Wang, Xiaoguang Chen, Chunhong Yan

**Affiliations:** 1 Institute of Materia Medica, Chinese Academy of Medical Sciences and Peking Union Medical College, Beijing, China; 2 Georgia Cancer Center, Augusta University, Augusta, GA USA; 3 Harbin Commercial University, Heilongjiang, China; 4 School of Pharmacy, Yantai University, Yantai, Shandong Province China; 5 Department of Pathology, Medical College of Georgia, Augusta University, Augusta, GA USA; 6 Department of Radiation Oncology, Ohio State University, Columbus, OH USA; 7 Department of Biochemistry and Molecular Biology, Medical College of Georgia, Augusta University, Augusta, GA USA

**Keywords:** Molecular biology, Cell biology

## Abstract

The amino acid antiporter system Xc^−^ is important for the synthesis of glutathione (GSH) that functions to prevent lipid peroxidation and protect cells from nonapoptotic, iron-dependent death (i.e., ferroptosis). While the activity of system Xc^−^ often positively correlates with the expression level of its light chain encoded by *SLC7A11*, inhibition of system Xc^−^ activity by small molecules (e.g., erastin) causes a decrease in the intracellular GSH level, leading to ferroptotic cell death. How system Xc^−^ is regulated during ferroptosis remains largely unknown. Here we report that activating transcription factor 3 (ATF3), a common stress sensor, can promote ferroptosis induced by erastin. ATF3 suppressed system Xc^−^, depleted intracellular GSH, and thereby promoted lipid peroxidation induced by erastin. ATF3 achieved this activity through binding to the *SLC7A11* promoter and repressing *SLC7A11* expression in a p53-independent manner. These findings thus add ATF3 to a short list of proteins that can regulate system Xc^−^ and promote ferroptosis repressed by this antiporter.

## Introduction

System Xc^−^ is an amino acid antiporter that mediates the exchange of extracellular cystine and intracellular glutamtate across the plasma membrane [1, 2]. Because cysteine reduced from cystine is the rate-limiting substrate for the synthesis of the antioxidant glutathione (GSH) [2], inhibition of system Xc^−^-mediated cystine import by small molecules (e.g., erastin) causes depletion of intracellular GSH and subsequent iron-dependent lipid peroxidation, which in turn leads to a form of nonapoptotic, iron-dependent cell death referred to as ferroptosis [3, 4]. Ferroptosis is regulated by a network revolving around glutathione peroxidase 4 (GPX4) that can reduce lipid peroxides at the expense of GSH, and thus GPX4 inhibitors (e.g., (1S, 3R)-RSL3, or RSL3 in short) are another major class of small molecules that can induce ferroptosis [5–7]. While ferroptosis has been linked to neurological disorders and ischemic injury in animal models [7], emerging evidence supports important roles that ferroptosis plays in tumor suppression and cancer resistance to therapy [8–12]. As the light chain encoded by *SLC7A11* is a subunit unique to system Xc^−^, the *SLC7A11* expression level often positively correlates with the activity of the antiporter [2]. Indeed, repression of *SLC7A11* expression by the tumor suppressor p53 or the histone deubiquitinating enzyme BAP1 inhibits cystine uptake and promotes ferroptosis [8, 9]. However, the mechanism by which *SLC7A11* expression is regulated remains largely unknown.

Activation transcription factor 3 (ATF3) is a member of the ATF/CREB family of transcription factors, and its expression is rapidly induced by a wide range of cellular stresses, including DNA damage, oxidative stress, and cell injury [13]. ATF3 contains a basic-region leucine-zipper (bZIP) domain that binds to the ATF/CREB *cis*-regulatory element or the AP-1 element [14], and as a result, can either repress or activate transcription depending on cell context. Although ATF3 can bind to more than 20,000 genome sites containing the ATF/CREB or the AP-1 element [14], only a small number of genes have been characterized so far as direct transcriptional targets of ATF3. In addition to its family members, ATF3 can also interact with other proteins (e.g., p53 and Tip60) and regulates their activities independent of its transcriptional activity [15, 16]. ATF3 has been shown to be involved in many pathological conditions such as cancer [17, 18], infections/inflammation [19–22], diabetes [23], and ischemic injury of hearts, livers, or brains [24, 25], highlighting the importance of understanding the exact role of ATF3 in the cellular stress response. Here we report that ATF3 could suppress system Xc^−^ and predispose cells to ferroptosis by repressing *SLC7A11* expression.

## Materials and methods

### Cell culture and reagents

HT1080 cells expressing ATF3 and empty vector were generated previously [26]. Retinal pigment epithelial (RPE) cells immortalized with hTERT were obtained from Dr. Todd Waldman [27]. To generate *ATF3*-knockout cells, a target sequence (5′-AAAATGATGCTTCAACACCC-3′) spanning the ATF3 start codon was inserted into pSpCas9(BB)-2A-puro, and used to transfect RPE and U2OS cells as described [28]. *ATF3*-knockout HCT116 cells were generated using rAAV-mediated homologous recombination as described previously [14]. These cells were cultured in media supplemented with 10% fetal bovine serum. We used DMEM for HT1080, RPE, DU145, and U2OS cells, RPMI 1640 for PC3 cells, and McCoy’s 5A for HCT116 cells. Erastin, RSL3, ferrostatin-1, BSO, Z-VAD-FMK, and FIN56 were purchased from Cayman Chemical. N-acetylcysteine (NAC) was purchased from Thermo Fisher Scientific. Other reagents were obtained from Sigma-Aldrich.

### Cell viability assays

Cells treated in 24-well plates were trypsinized, and stained with 0.2% of Trypan blue. Viable cells were then counted using a hemocytometer under a microscope. In some experiments, cell viability was assessed by MTT assays as described [29].

### *SLC7A11* promoter cloning and mutagenesis

The human *SLC7A11* promoter (−678 to +8) was PCR amplified from genomic DNA derived from HT1080 cells using a forward primer (5′-ATGGTACCTCTGGAGTCCTGGTGAATTTTG-3′) and a reverse primer (5′-TAACTCGAGACAAACCAGCTCAGCTTCCT-3′). After purification, the DNA fragment was digested with KpnI and XhoI, and cloned into the pGL3-Basic vector following a standard cloning protocol. To generate promoter constructs with BS-1 (m1) or BS-2 (m2) mutations, overlapping PCR was performed using the wild-type promoter as the template, and the forward/reverse primer coupled with a pair of complementary primers spanning the BS-1 or the BS-2 site. These primers were: m1-forward (5′-TTCTCATGTGGCGGGTGCAAACCTGG-3′), m1-reverse (5′-CCAGGTTTGCACCCGCCACATGAGAAG-3′); m2-forward (5′-CCTGGAGAATTTGCACCCTCATTTAGCTGTAG-3′), m2-reverse (5′-CTACAGCTAAATGAGGGTGCAAATTCTCCAGG); the mutated nucleosides were underlined. Double mutation were generated by using m1 as the template coupled with the m2 primer pair. The mutant promoter fragments were cloned into pGL3-Basic at KpnI and XhoI sites. To generate the promoter construct with a deletion of the predicted p53-BS, reverse PCR was performed using the wild-type promoter construct as the template and a forward primer (5′-TGCCTGTCACACCAACTTAC-3′) and a reverse primer (5′-ATGAGGAAGCTGAGCTGGTT-3′). The PCR product was digested with 5 units of DpnI, purified, and ligated with T4 DNA ligase for transformation. The sequences of the wild-type and mutant promoters were confirmed by DNA sequencing.

### Transfections and reporter assays

Transfections were carried out using Lipofectamine 2000 (Invitrogen) according to a protocol provided by the manufacturer. For reporter assays, cells seeded in 24-well plates were transfected with 100 ng of pGL3 or pGL3-pSLC7A11, 5 ng of pRL-CMV, and 400 ng of pCG-ATF3 or pCG, or 400 ng of p53. After 48 h, cells were lysed, and the reporter activity was measured using the Dual-Luciferase Reporter Assay System (Promega) following the recommendation.

### Lentiviral infections

To generate the lentiviral vector expressing ATF3, the ATF3 cDNA was PCR amplified and cloned into pCDH-CMV-MCS-EF1-Puro at NheI and BamHI sites. The lentivectors (3 μg) were transfected into 293FT cells plated in a 100 mm dish along with 3 μg of pLP1, 3 μg of pLP2, and 3 μg of pLP/VSVG. Supernatants were harvested 2 days later and used to infect cells along with 4 μg/ml of polybrene. The *SLC7A11*-expressing lentiviral vector (pLOC-SLC7A11) and its control vector (pLOC-RFP) were previously described [30] and provided by Dr. Nicholas Clemons.

### Chromatin immunoprecipitation assays

These were carried out as described previously [14]. We used an anti-ATF3 antibody (Santa Cruz, sc-188) or normal rabbit IgG to pull down sheared chromatin. Immunoprecipitated DNA was purified using the QIAquick PCR Purification Kit (Qiagen), and quantitated using real-time PCR. The primers used were as follows: SLC7A11, forward 5′-TTGAGCAACAAGCTCCTCCT-3′, reverse 5′-CAAACCAGCTCAGCTTCCTC-3′; ATF3, forward 5′-TGGCAACACGGAGTAAACGAC-3′, reverse 5′AGAGAAGAGAGCTGTGCAGTGC; RNF-ex, forward 5′-GGCTGGATTTTTGCAAGTTGA-3′, reverse 5′-TTGACGCCTCCAGCATCTG-3′.

### DNA affinity precipitation assays

Nuclear extracts were prepared as described previously [31]. 100 μg of nuclear extracts or 0.25–1 μg of recombinant ATF3 [16] were incubated with 2 μg of annealed biotin-labeled or unlabeled oligonucleotide (biotin-5′-CATGTGGCTTGATGCAAACCTGGAGAATTTGCATCATCATTTAGC-3′) in a buffer containing 25 mM Tris-HCl, pH7.9, 2 mM EDTA, 1 mM EGTA, 0.1% NP-40, 100 mM NaCl, 1 mM DTT, and 5% glycerol at 4 °C for 1 h, and then at 4 °C for 2 h with addition of 15 μl of Dynabeads M-280. The beads were washed with the incubation buffer supplemented with 150 mM NaCl and 0.5% NP-40 for five times. Bound proteins were eluted by boiling in 20 μl of SDS loading buffer, and subjected to western blotting. For competition assays, ATF3 was incubated with 50 μg of unlabeled oligonucleotide along with 2 μg of labeled probe.

### Glutamate release assays

The Glutamate-Glo Assay kit (Promega) was used to measure the amount of glutamate released into condition medium. Cells (2 × 10^5^) plated in six-well plates were washed with PBS twice, and incubated in glutamine-free medium in the presence/absence of 10 μM of erastin for 1 h. To measure the glutamate level, 50 μl of condition medium was transferred to a 96-well plate, and mixed with 50 μl of a reaction mixture containing glutamate dehydrogenase, NAD, reductase, pro-luciferin, and luciferin detection solution following the manufacturer’s protocol. The plate was shaken for 30–60 s and incubated at room temperature for 1 h, and luminescence was measured with SpectraMax L (Molecular Devices). The glutamate level was first calculated in reference to a glutamate standard curve, and then normalized to the total cell number determined by MTT assays at the end of the experiment.

### GSH assay

The Glutathione Assay Kit (Cayman Chemical) was used for these experiments. Cells (2 × 10^5^) in six-well plates were treated with or without 10 μM of erastin for 5 h, scraped into 500 μl of 10 mM phosphate buffer containing 1 mM EGTA, and then lysed by sonication. After centrifugation, supernatants were deproteinated by incubating with 500 μl of 10% metaphosphoric acid at room temperature for 5 min, and then centrifuged for 3 min at 4000 rpm. The resulted supernatants were mixed with 50 μl of 4 M triethanolamine. 50 μl of each sample was then transferred to a 96-well plate, and incubated with 150 μl of Assay Cocktail containing the Ellman’s reagent (5,5′-dithio-bis- 2-(nitrobenzoic acid) or DTNB) at room temperature for 25 min. The absorbance at 405 nm was measured, and used to calculate the GSH amount in reference to a GSH standard curve.

### Lipid peroxidation detection

Cells (2 × 10^5^) in six-well plates were incubated with 1 ml of fresh medium containing 5 μM of BODIPY 581/591 C11 (Invitrogen) for 20 min. Cells were then trypsinized, washed, and resuspended in 0.5 ml of PBS for flow cytometry analysis. A minimum of 20,000 cells were analyzed per condition.

### Quantitative RT-PCR

Total RNA was extracted from cells using TRIzol (Invitrogen). 2 μg of total RNA was then reverse transcribed using the iScript Advanced cDNA kit for RT-qPCR (Bio-Rad), and subjected to real-time PCR using the SYBR Green reagents (Bimake). The sequences of the primers used for real-time PCR were: SLC7A11, forward 5′-TCATTGGAGCAGGAATCTTCA-3′, reverse 5′-TTCAGCATAAGACAAAGCTCCA-3′; ATF3, forward 5′-GTGCCGAAACAAGAAGAAGG-3′, reverse 5′-TCTGAGCCTTCAGTTCAGCA-3′; CHAC1, forward 5′-GAACCCTGGTTACCTGGGC-3′, reverse 5′-CGCAGCAAGTATTCAAGGTTGT-3′; ASNS, forward 5′-CTGTGAAGAACAACCTCAGGATC-3′, reverse 5′-AACAGAGTGGCAGCAACCAAGC-3′; p21, forward, 5′-CTGGAGACTCTCAGGGTCGAAA-3′, reverse 5′-GATTAGGGCTTCCTCTTGGAGAA-3′; MMP-2, forward 5′-CCCACTGCGGTTTTCTCGAAT-3′, reverse 5′-CAAAGGGGTATCCATCGCCAT-3’; β-actin, forward 5′-TCCATCATGAAGTGTGACG-3′, reverse 5′-TACTCCTGCTTGCTGATCCAC-3′.

### Western blotting

These were performed as described previously [15]. Briefly, cells were lysed in RIPA buffer containing 50 mM Tris-HCl, pH 7.4, 1% Nonidet P-40, 0.25% sodium deoxycholate, 150 mM NaCl, 1 mM EDTA, 1 mM PMSF, 1 mM NaF, 1 mM Na_3_VO_4_, and protease inhibitor cocktail (Roche) for 30 min, and subjected to SDS-PAGE for western blotting. The following antibodies were used: SLC7A11 (1:1000, #12691) from Cell Signaling, ATF3 (1:1000, sc-188) and p53 (1:1000, sc-6243) from Santa Cruz, and β-actin (1:10000, A5441) from Sigma.

## Results

### ATF3 promotes ferroptosis induced by erastin

**Fig. 1:**
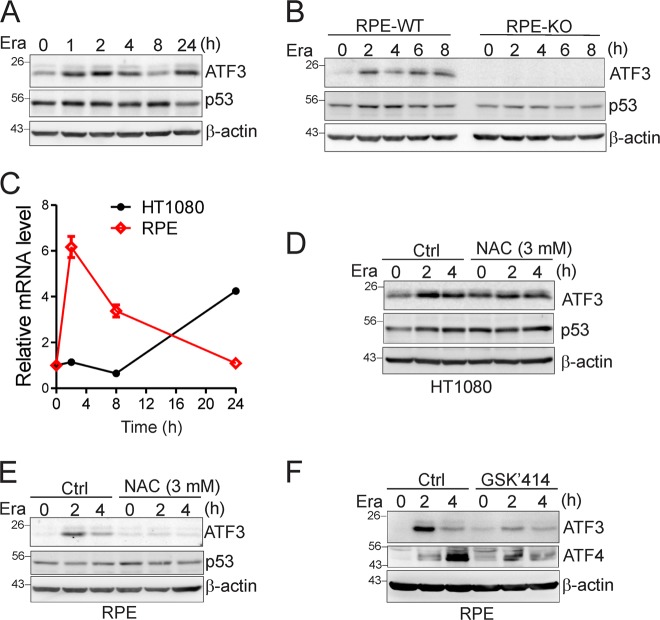
Metabolic stress induces *ATF3* expression. **a**, **b** HT1080 and RPE cells were treated with 5 µM of erastin for different time as indicated, and subjected to western blotting for *ATF3* and p53 expression. **c** Indicated cells were treated with 5 μM of erastin for different time and subjected to qRT-PCR to measure the *ATF3* mRNA level. The data are presented as mean ± SD. **d**, **e** HT1080 and RPE cells treated with 5 μM of erastin with or without 3 mM of NAC were subjected to western blotting. **f** RPE cells were pretreated with or without 1 µM of GSK2606414 (GSK′414) for 30 min, and then treated with erastin (5 µM) for western blotting

A recent finding that the *ATF3* mRNA level was increased during erastin-induced ferroptosis [4] prompted us to hypothesize that ATF3 may play a role in the regulation of this important form of cell death. Indeed, we found that erastin could induce *ATF3* expression in as short as 1 h (Fig. 1a–c). The induction of *ATF3* expression was transient in the immortalized normal RPE cell line (Fig. 1b–c), probably due to transcriptional autorepression [32]. NAC, the precursor of intracellular antioxidant GSH, could inhibit ATF3 induction by erastin (Fig. 1d, e), suggesting that the Nrf2-mediated antioxidant pathway activated by FIN-induced redox imbalance [20] might mediate ATF3 induction by these agents. Indeed, while *ATF3* is a known oxidative stress-responsive gene [33], Nrf2 has been shown to bind to the *ATF3* promoter and mediate *ATF3* expression induced by reactive oxygen species (ROS) [34]. Similarly, while *ATF3* expression can be induced by the endoplasmic reticulum (ER) stress via the PERK/ATF4-mediated pathway [35], *ATF3* induction by erastin was impaired when the cells were pretreated with an PERK inhibitor GSK 2606414 (Fig. 1f), suggesting that erastin-induced ER stress [4] might contribute to *ATF3* induction as well. However, although *ATF3* was a p53 target gene in a certain contexts [36, 37], the observations that the p53 level was barely elevated by erastin (Fig. 1a, b, d, e) suggest that p53 was not involved in *ATF3* induction by erastin.

**Fig. 2:**
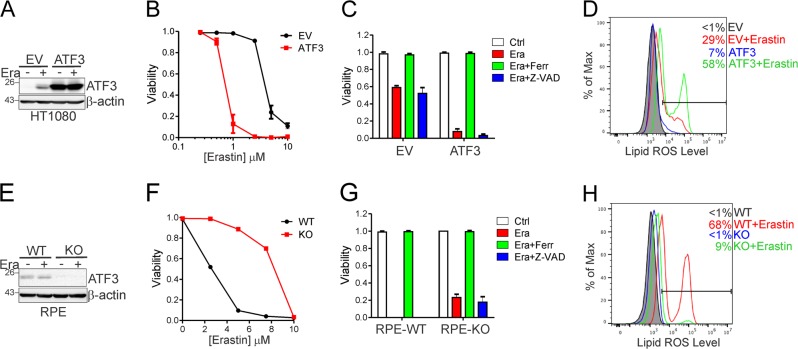
ATF3 promotes ferroptosis induced by FINs. **a** HT1080 cells expressing ATF3 or transfected with the empty vector (EV) were treated with 2.5 μM of erastin for 24 h, and subjected to western blotting. **b** Viable HT1080 cells treated with erastin for 24 h were counted after stained with Trypan blue. **c** Viable HT1080 cells were counted after treatments with erastin (5 μM), Ferr-1 (2 μM), and/or Z-VAD-FMK (10 μg/ml) for 24 h. **d** HT1080 cells treated with/without erastin for 6 h were stained with C11-BODIPY, and subjected to flow cytometry analysis. **e** ATF3-knockout (KO) or -wild-type (WT) RPE cells were treated with 2.5 μM of erastin for 24 h for western blotting. **f** Viable RPE cells treated with erastin for 24 h were counted. **g** Viable RPE cells were counted after treated with erastin (5 μM), Ferr-1 (2 μM), and/or Z-VAD-FMK (10 μg/ml) for 24 h. **h** RPE cells treated with/without erastin for 6 h were stained with C11-BODIPY for flow cytometry analysis

Ferroptosis inducers (FINs) can readily induce HT1080 cells to undergo ferroptosis—an event characterized by lipid peroxidation that can be alleviated by ferrostatin-1 (Ferr-1) [3]. Intriguingly, *ATF3* expression sensitized HT1080 cells to erastin-induced death (Fig. 2a, b) that could be rescued by Ferr-1 but not Z-VAD-FAM (Fig. 2c), indicating that ATF3 promoted erastin-induced ferroptosis. Consistent with this effect, *ATF3* expression promoted erastin-induced elevation of the lipid peroxide level in these cells (Fig. 2d). These effects were not caused by clonal variation, as we saw similar effects in an unselected cell population expressing *ATF3* via lentiviral infections (Supplementary Fig. S1A). Although overexpression of an ectopic gene may trigger ROS production leading to cell death, it was highly unlikely that the observed ATF3-mediated effects were merely a consequence of gene overexpression as overexpression of an ATF3 mutant (ATF3-ΔBR) that lacks its DNA-binding domain (the basic region, or BR), and thus does not regulate gene transcription, failed to sensitize HT1080 cells to erastin (Supplementary Fig. S2). To corroborate these results, we generated *ATF3*-knockout (KO) RPE cells with the CRISPR/Cas9 tool (Fig. 2e). While the normal epithelial cells were sensitive to erastin (Fig. 2f), knockout of *ATF3* expression dramatically suppressed erastin-induced ferroptosis and lipid peroxidation (Fig. 2f–h). ATF3 promoted erastin-induced ferroptosis in other cell lines (e.g, DU145 and U2OS) as well (Supplementary Fig. S1B, S1C). Taken together, our results indicate that ATF3 can promote ferroptosis induced by erastin. Of note, *ATF3* expression promoted, while *ATF3* knockout suppressed ferroptosis induced by the GPX4 inhibitor RSL3—a type 2 FIN—as well (Supplementary Fig. S3A, S3B). However, ATF3 had a negligible effect on cell sensitivity to FIN56, a type 3 FIN, which induces ferroptosis by depleting CoQ10 and GPX4 [7, 38] (Supplementary Fig. S3D, 3E).

### ATF3 suppresses system Xc^−^

**Fig. 3:**
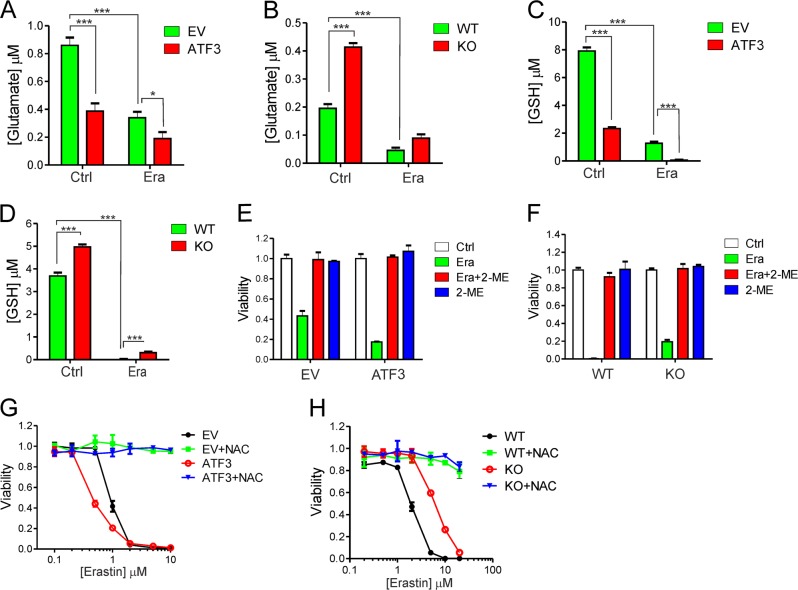
ATF3-mediated suppression of system Xc^−^ contributes to the promotion of ferroptosis. **a**, **b** HT1080 and RPE cells were treated with or without 10 μM of erastin for 1 h, and the amount of glutamate released into culture medium was measured. **c**, **d** Indicated cells treated with 10 μM of erastin for 5 h were lysed, and the intracellular GSH level was measured. **e** Viable HT1080 cells after treatments with erastin (1 μM) and/or 2-ME (20 μM) were counted. **f** Viable RPE cells treated with erastin (5 μM) and/or 2-ME (20 μM) were counted. **g**, **h** Cells were treated with erastin and/or NAC (3 mM), and subjected to MTT assays. The data are presented as mean ± SD. *p < 0.05; ***p < 0.001, Student’s *t*-test

System Xc^−^-mediated cystine uptake crucial for GSH synthesis is a major mechanism that blocks lipid peroxidation and suppresses ferroptosis [5, 6]. To understand the mechanism by which ATF3 promotes ferroptosis, we determined the activity of system Xc^−^ by measuring the amount of glutamate released into cultured media [4]. While erastin inhibited glutamate release as expected [4], *ATF3* expression caused a decrease in the amount of released glutamate even in the absence of erastin (Fig. 3a). Conversely, *ATF3* KO promoted glutamate release (Fig. 3b). The effects of ATF3 on glutamate release were consistent with the results that *ATF3* expression increased while KO decreased the intracellular GSH level (Fig. 3c, d), arguing for the notion that ATF3 suppresses system Xc^−^. Thus, ATF3 might promote ferroptosis through suppressing system Xc^−^.

### ATF3 promotes ferroptosis through regulating system Xc^–^

2-Mercaptoethanol (2-ME) can reduce cystine to cysteine extracellularly, and thus bypass system Xc^−^ to supply cysteine for GSH synthesis. If suppression of system Xc^−^ is the main mechanism by which ATF3 promotes ferroptosis, bypassing system Xc^−^ by treating cells with 2-ME would alleviate the effects of ATF3 on ferroptosis. Indeed, 2-ME rescued ferroptosis induced by erastin in *ATF3*-expressing HT1080 cells (Fig. 3e). Similarly, erastin-induced ferroptosis in RPE cells was also rescued (Fig. 3f). Of note, it is unlikely that the general/nonspecific reducing activity of 2-ME resulted in the observed rescuing effect, as 2-ME did not recue ferroptosis induced by cystine deprivation (Supplementary Fig. S4). We also treated the cells with NAC, which can convert into cysteine, thereby bypassing system Xc^−^. Again, bypassing system Xc^−^ using NAC abolished ferroptosis induced by erastin in both HT1080 and RPE cells (Fig. 3g, h). These results thus support our hypothesis whereby ATF3 promotes erastin-induced ferroptosis through suppressing system Xc^−^.

### ATF3 binds to and represses the *SLC7A11* promoter

**Fig. 4:**
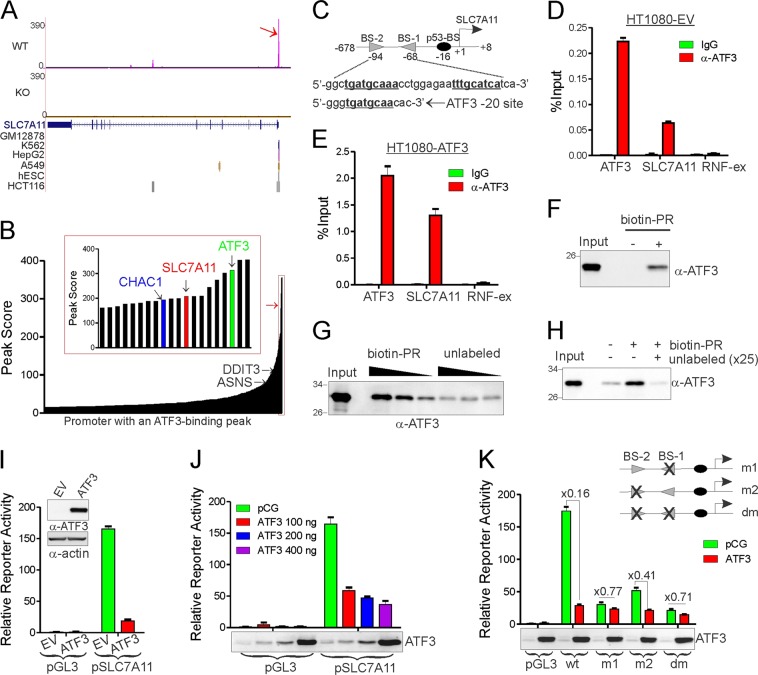
ATF3 binds to and represses the *SLC7A11* promoter. **a** A UCSC genome browser view of the ATF3-binding peak in the human *SLC7A11* promoter. The ChIP-seq data previously reported [14] were reanalyzed and compared with the data from the ENCODE project. WT and KO, ATF3-wildtype and -knockout cells, respectively. **b** Peak scores of ATF3-binding peaks localized in promoters (defined as regions +/− 1 kb relative to transcription start sites) are depicted. Insert indicates top promoters with highest peak scores. **c** The sequence of the center of the ATF3-binding peak in the human *SLC7A11* promoter is shown. The locations of BS-1, BS-2, and p53-BS relative to the transcription start site are indicated. **d**, **e** HT1080 cells were subjected to ChIP assays with an ATF3-specific antibody, and the amount of precipitated DNA was quantitated by real-time PCR. **f** A biotin-labeled oligonucleotide containing the BS-1 and BS-2 sites (biotin-5′-CATGTGGCTTGATGCAAACCTGGAGAATTTGCATCATCATTTAGC-3′) was incubated with nuclear extracts prepared from HT1080-ATF3 cells. Bound proteins were pulled down using Dynabeads, washed, and subjected to western blotting. **g** The biotin-labeled and unlabeled oligonucleotides were incubated with recombinant ATF3 for pulldown assays as in **f**. **h** The biotin-labeled oligonucleotide were incubated with recombinant ATF3 with or without an excess amount of unlabeled oligonucleotide for pulldown assays. **i** The human *SLC7A11* promoter (pSLC7A11) was cloned into pGL3, and transfected into HT1080 cells for dual luciferase assays. **j** HT1080 cells were transfected with the indicated reporters with or without increasing amounts of ATF3 for dual luciferase assays. **k** The *SLC7A11* promoters with point mutations in the BS-1 (5′-TTGCATCA-3′ to 5′-TTGCACCC-3′) and/or BS-2 site (5’-TGATGCAA-3’ to 5′-GGGTGCAA-3′) were transfected with/without ATF3 into HT080 cells for dual luciferase activity assays

As the *SLC7A11* expression level positively correlates with the system Xc^−^ activity [2], we tested if ATF3 suppresses system Xc^−^ by downregulating *SLC7A11* expression. Intriguingly, our genome-wide ChIP-seq analysis [14] revealed a very strong ATF3-binding peak in a *SLC7A11* promoter region proximal to the transcription start site (TSS) (Fig. 4a, arrow). This ATF3-binding peak was also found in almost all of the cell lines examined by the ENCODE project, including human embryonic stem cells (Fig. 4a), suggesting that ATF3 likely strongly binds to the *SLC7A11* promoter and regulates *SLC7A11* transcription. It is important to note that the *SLC7A11* promoter is on the list of top ten promoters with highest ATF3-binding scores (Fig. 4b). As a comparison, the binding score of *DDIT3* or *CHOP*—a gene well-characterized as an ATF3 target [39]—was much lower than that of *SLC7A11* (Fig. 4b). The ATF3-binding peak in the *SLC7A11* promoter spans a region containing two adjacent sites (BS-1 and BS-2) that are in opposite orientation but have the same sequence (5′-TGATGCAA-3′) as the –20 site that was previously characterized as a functional ATF3-binding site in the *ATF3* promoter and known to be responsible for its autorepression [32] (Fig. 4c), suggesting that ATF3 likely binds to the *SLC7A11* promoter at these two sites. Indeed, we carried out ChIP assays using HT080 cells, and confirmed that ATF3 strongly bound to this region in spite of the very low basal ATF3 level in the cells (Fig. 4d). We used the *ATF3*-20 site (ATF3) and a region spanning a *RNF43* exon (RNF-ex) as the positive and the negative control, respectively, based on our previous report [14]. Overexpressing *ATF3* in HT080 cells significantly increased the amount of ATF3 bound to the *SLC7A11* promoter (Fig. 4e), further demonstrating the specificity of the binding. We also carried out DNA affinity precipitation assays, and confirmed that ATF3 bound to an oligonucleotide spanning this region (Fig. 4f). Moreover, while recombinant ATF3 also bound to the oligonucleotide (Fig. 4g), such binding was competitively blocked by an excess amount of unlabeled oligonucleotide (Fig. 4h), suggesting that ATF3 can directly bind to the *SLC7A11* promoter.

To determine whether ATF3 binding to the *SLC7A11* promoter is functional, we cloned the human *SLC7A11* promoter for luciferase reporter activity assays. While the *SLC7A11* promoter exhibited a dramatically-reduced activity in HT1080 cells stably expressing *ATF3* (Fig. 4i), transient *ATF3* expression also significantly decreased the *SLC7A11* promoter activity in a dose-dependent manner (Fig. 4j). Importantly, mutating either BS-1 or BS-2 dramatically alleviated ATF3-mediated repression of the *SLC7A11* promoter (Fig. 4k), indicating that ATF3 bound to both BS-1 and BS-2 sites to repress the *SLC7A11* promoter. Of note, mutating either BS-1 or BS-2 site largely decreased the basal activity of the promoter (Fig. 4k)—a result arguing for a model whereby ATF3 represses the *SLC7A11* promoter by competing for BS-1/BS-2 binding with transcriptional activators (see more in “Discussion”).

### P53 is not required for ATF3-mediated repression of *SLC7A11* promoter

**Fig. 5:**
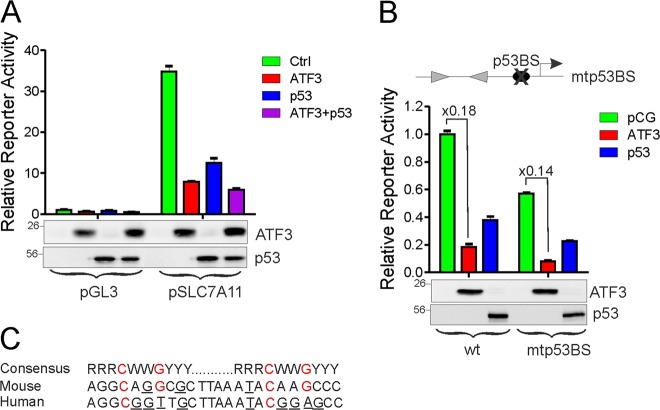
ATF3 represses the *SLC7A11* promoter independent of p53. **a** p53-null PC3 cells were transfected with the *SLC7A11* promoter reporter with or without ATF3/p53 for dual luciferase activity assays. **b** The p53-binding site (p53-BS) was deleted from the *SLC7A11* promoter. The mutant promoter (mtp53BS) was transfected into PC3 cells with or without ATF3 or p53 for dual luciferase activity assays. **c** The sequence of the predicted p53-binding site in the human *SLC7A11* promoter was compared with that in the mouse promoter and the consensus p53-binding sequence. Red color marks the four nucleotides crucial for p53 binding. The nucleotides that do not match the consensus sequence are underlined. The data are presented as mean ± SD

Previously, p53 was shown to repress *SLC7A11* transcription by binding to a consensus site (p53-BS) proximal to BS-1 and BS-2 sites (Fig. 4c) [8]. As ATF3 can interact with p53 and colocalize with p53 at promoters/enhancers to regulate transcription [14, 15], it is probable that ATF3 might regulate the *SLC7A11* promoter through interacting with p53. However, ATF3 retained its *SLC7A11*-repressing activity in p53-null PC3 cells (Fig. 5a). In line with these results, while the amount of ATF3 bound to the promoter of an ATF3- and p53-coregulated gene *MSI2* [14] was decreased, knockout of p53 did not impair ATF3 binding to the *SLC7A11* promoter (Supplementary Fig. S5). Moreover, ATF3 repressed a mutated *SLC7A11* promoter where p53-BS was deleted (mtp53BS) to the same extent as the wild-type promoter (Fig. 5b). Surprisingly, p53 also repressed the mutant promoter (Fig. 5b). While p53 may repress transcription without binding to a consensus site [40, 41], it is worth noting that, unlike in the mouse promoter [8], two of the four nucleotides crucial for p53 binding (marked in red) in the human *SLC7A11* promoter don’t match that of the consensus site (Fig. 5c).

### ATF3 represses *SLC7A11* expression

**Fig. 6:**
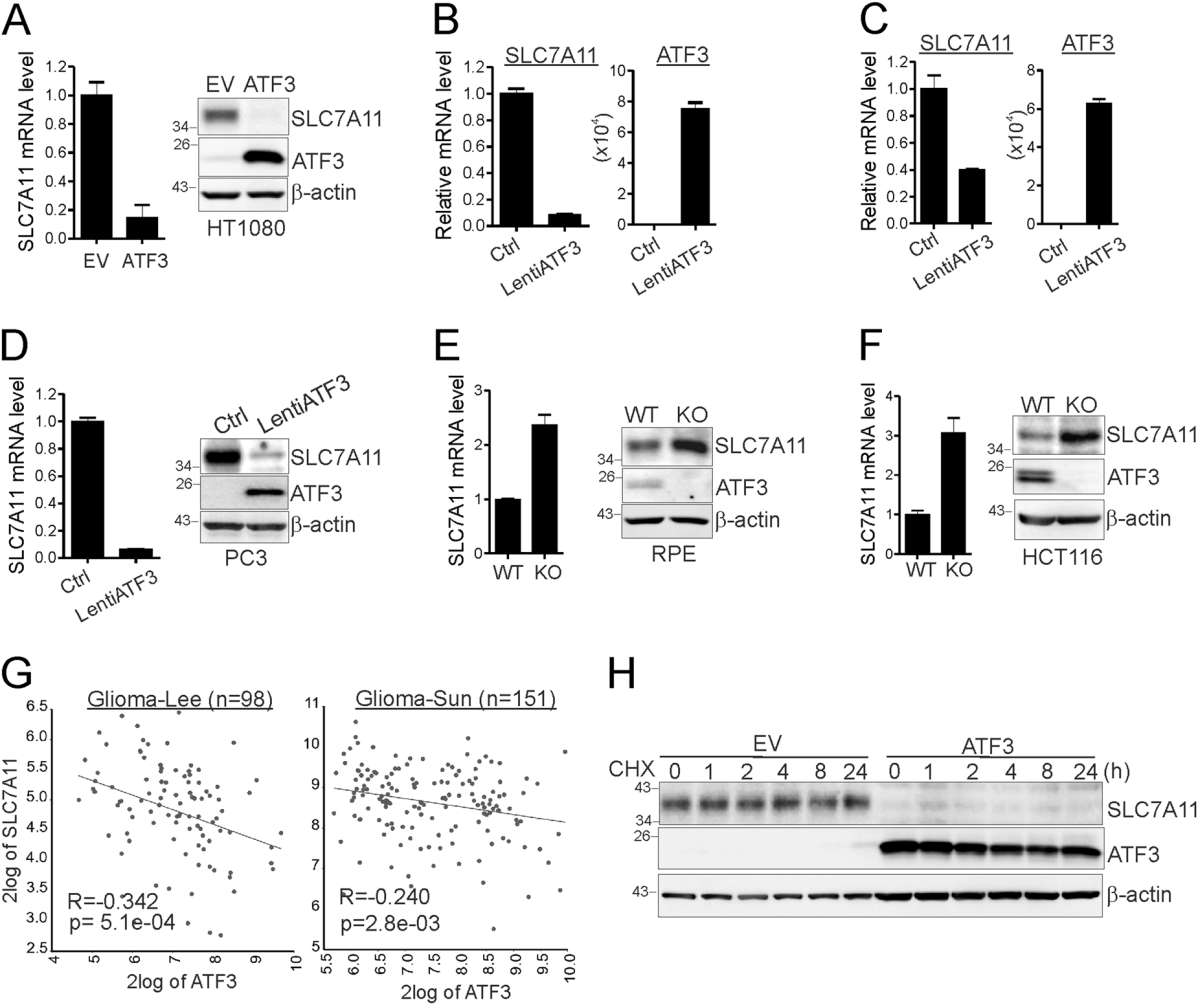
ATF3 represses *SLC7A11* expression. **a** HT1080 cells were subjected to qRT-PCR and western blotting for *SLC7A11* expression. **b**, **c** HT1080 (**b**) and RPE (**c**) cells were infected with LentiATF3 or control viruses for qRT-PCR assays. **d** PC3 cells infected with LentiATF3 was subjected to qRT-PCR and western blotting for *SLC7A11* expression. **e** The *SLC7A11* expression level was determined in ATF3-wiltype (WT) and -knockout (KO) RPE cells. **f** *ATF3*-knockout (KO) HCT116 cells generated via rAAV-mediated gene targeting were subjected to qRT-PCR and western blotting for *SLC7A11* expression. **g** The correlation between *ATF3* and *SLC7A11* expression in two cohorts of glioma patients was analyzed using the R2 Genomics Analysis and Visualization Platform. **h** HT1080 cells were treated with cycloheximide (CHX) for western blotting.The data are presented as mean ± SD

Consistent with the results that ATF3 repressed the *SLC7A11* promoter, both the *SLC7A11* mRNA and protein levels were dramatically decreased in the *ATF3*-expressing cells compared to the control HT1080 cells (Fig. 6a) - an effect unlikely caused by clonal variation as lentiviral-mediated *ATF3* expression repressed *SLC7A11* expression in HT1080 (Fig. 6b) and RPE cells (Fig. 6c) as well. ATF3 also decreased the *SLC7A11* level in p53-null PC3 cells (Fig. 6d), in line with the notion that ATF3 represses *SLC7A11* expression independent of p53. Conversely, knockout of *ATF3* expression in RPE cells caused an increase in the *SLC7A11* level (Fig. 6e). This effect was not due to nonspecific gene targeting by the ATF3-sgRNA, as knockout of *ATF3* expression using a distinct, rAAV-based approach [14] also increased the *SLC7A11* expression level (Fig. 6f). To further support the notion that ATF3 represses *SLC7A11* expression, we analyzed publicly available gene expression datasets and found that the *ATF3* expression level inversely correlated with the *SLC7A11* level in two cohorts of glioma patients (Fig. 6g). This correlation is of significance given that *SLC7A11* overexpression found in glioma cells has been shown to promote the growth of the brain tumors [42]. As the SLC7A11 protein had a relatively long half-life, it was not surprising that ATF3 did not affect the stability of this protein (Fig. 6h).

### Stress-induced ATF3 does not regulate *SLC7A11* expression

**Fig. 7:**
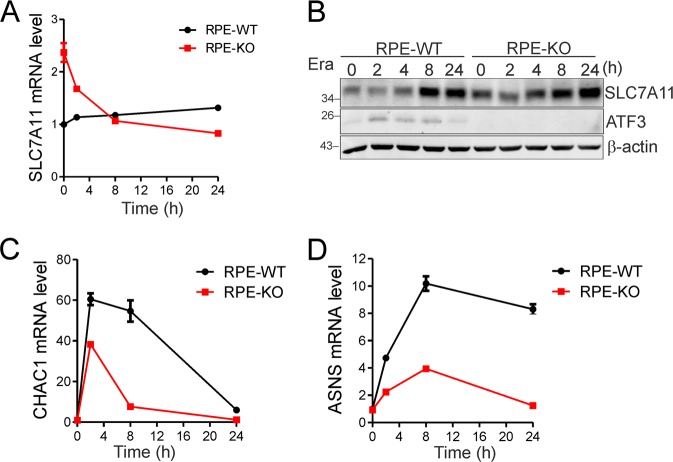
Erastin-induced ATF3 does not repress *SLC7A11* induction but promote CHAC1 and ASNS induction. **a**, **b** RPE cells treated with 5 μM of erastin were subjected to qRT-PCR (A) and western blotting (B). **c**, **d** *CHAC1* and *ASNS* expression levels in ATF3-wildtype (WT) and –KO RPE cells were determined by qRT-PCR

Erastin can induce *SLC7A11* expression as an adaptive response to system Xc^−^ inhibition [4]. Indeed, erastin induced a slight increase of the *SLC7A11* mRNA level (Fig. 7a), but profound accumulation of the SLC7A11 protein (Fig. 7b), in RPE cells, suggesting that erastin might induce an adaptive cellular response (e.g., inducing expression of the heat shock protein HSPA5 [10]) to protect SLC7A11 from degradation. To our surprise, knockout of *ATF3* expression in RPE cells caused a decrease in the *SLC7A11* mRNA level after erastin treatments (Fig. 7a). However, because erastin only slightly increased the *SLC7A11* mRNA level (Fig. 7a) and since the half-life of SLC7A11 protein was relatively long (Fig. 7b), the SLC7A11 protein level remained higher in the knockout cells as compared to the wild-type cells at all of the time points during erastin treatments (Fig. 7b), which would ensure higher system Xc^−^ activity in the KO cells. Intriguingly, while ATF3 could bind to the *CHAC1* and the *ASNS* promoter (Fig. 4b), ATF3 promoted erastin-induced expression of these two genes [4] (Fig. 7c, d). While the role of neither CHAC1 nor ASNS in the regulation of ferroptosis has been established [4], our results suggest that ATF3 more likely controls the basal *SLC7A11* level and predispose cells to a state sensitive to ferroptosis.

### *SLC7A11* expression antagonizes ATF3-mediated promotion of ferroptosis

**Fig. 8:**
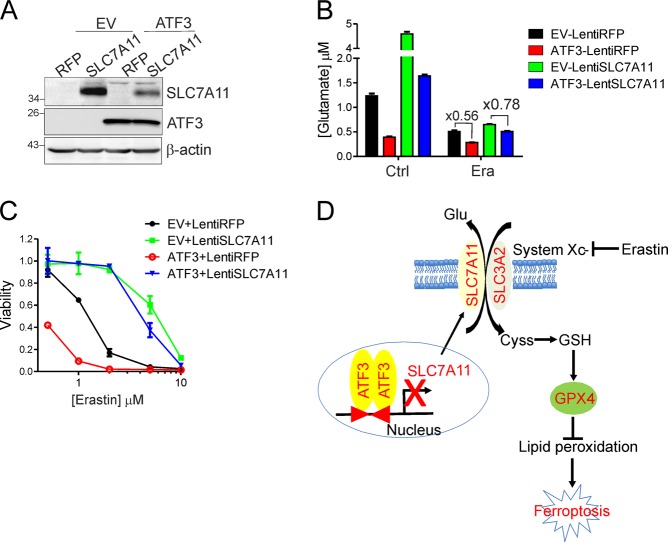
*SLC7A11* overexpression antagonizes ATF3-promoted ferroptosis. **a** HT1080 cells were infected with *SLC7A11*-expressing lentiviruses or control viruses (RFP) for western blotting. **b** The amount of glutamate in the condition media from the infected HT1080 cells treated with or without erastin (10 μM) was measured. **c** Infected cells were treated with erastin, and cell viability was measured by MTT assays. **d** Schematic presentation of a model whereby ATF3 represses *SLC7A11* expression to suppress system Xc^-^, and thereby predispose cells to a state prone to ferroptosis

The results described above argue for that ATF3 represses *SLC7A11* expression to suppress system Xc^−^ and subsequently promote ferroptosis. If this hypothesis is true, restoring *SLC7A11* expression in *ATF3*-expressing cells would restore system Xc^−^ activity and consequently impair the capability of ATF3 to promote ferroptosis. Indeed, *SLC7A11* overexpression via lentiviral infections (Fig. 8a) restored the system Xc^−^ activity (evidenced by increased glutamate release) in *ATF3*-expressing cells (Fig. 8b). The reason why the *SLC7A11* level in *ATF3*-expressing cells was lower than that in the empty-vector control cells after lentiviral infections was unknown, but it might be attributable to the capability of ATF3 to suppress viral integration [43]. Nevertheless, while *SLC7A11* expression conferred resistance to erastin in both ATF3-expressing and control cells as expected, the difference in erastin sensitivity between these cells was diminished (Fig. 8c), indicating that *SLC7A11* expression counteracted ATF3-mediated ferroptosis induced by erastin. Our results thus demonstrate that ATF3 can promote erastin-induced ferroptosis by repressing *SLC7A11* expression and suppressing system Xc^−^.

## Discussion

Ferroptosis is a nonapoptotic form of cell death that can be induced by metabolic stress such as GSH depletion [7]. While system Xc^−^ mediated uptake of cystine is crucial for the maintenance of redox balance and the blockage of lipid peroxidation, how the system Xc^−^ activity is regulated remains largely unknown. Previously, CD44v/MUC1-C and BECN1 was shown to interact with SLC7A11 and regulate the stability of system Xc^−^ [44, 45]. While Nrf2 and ATF4 can bind to the *SLC7A11* promoter and activate its expression [46–48], binding of p53 to the *SLC7A11* promoter or BAP1-mediated epigenetic modifications of the promoter represses *SLC7A11* transcription [8, 9]. In this report, we have demonstrated that ATF3 is another major SLC7A11 repressor. ATF3 strongly bound to the *SLC7A11* promoter, suppressed system Xc^−^, and promoted ferroptosis induced by erastin (Fig. 8d). Although ATF3 expression was induced by erastin, it is intriguing that ATF3 induction was more likely a mere response to redox imbalance caused by system Xc^−^ inhibition (Fig. 1d, e). In this regard, while both the *ATF3* and the *SLC7A11* promoter contain binding sites for Nrf2 that is activated by oxidative stress [34, 46], erastin-induced ATF3 did not appear to repress *SLC7A11* expression induced by redox imbalance. Rather, ATF3 contributed to erastin-induced *CHAC1* expression (Fig. 7c). Although CHAC1 was shown to mediate GSH degradation [49, 50], its role in ferroptosis remains controversial [4, 51]. Therefore, it is more likely that ATF3 predisposes cells to a state prone to ferroptosis through repressing *SLC7A11* expression. Our study thus adds ATF3 to a very short list of proteins that can regulate ferroptosis by suppressing system Xc^−^. Given that emerging evidence links ferroptosis to various pathological conditions (e.g., cancer and neural injury) [7] often involving induction or aberrant expression of ATF3 [13, 17], our finding suggests that ATF3 may regulate these events by promoting ferroptosis. It is worth noting that ATF3 is only structurally similar to ATF4 in the bZIP domain. In contrast to ATF3, ATF4 suppresses ferroptosis by transactivating *SLC7A11* and *HSPA5* [10, 48]. As *Atf3*^*−/−*^ mice, unlike *Atf4*^*−/−*^ mice, are developmentally normal [52], drugging ATF3 would be a better strategy than targeting ATF4 for harnessing ferroptosis to treat human diseases including cancer.

Intriguingly, ATF3 also appeared to promote RSL3-induced ferroptosis (Supplementary Fig. S3A, S3B). While ATF3-mediated suppression of system Xc^−^ resulted in a decrease in the intracellular GSH level (Fig. 3c), which could in turn sensitize the cells to RSL3-mediated GPX4 inhibition, bypassing system Xc^−^ with 2-ME or NAC indeed countered the effects of ATF3 on RSL3 sensitivity (Supplementary Fig. S3C). However, 2-ME/NAC only partly antagonized ATF3’s effects, suggesting that ATF3 could also promote RSL3-mediated ferroptosis via additional, unknown mechanism(s). Indeed, like ATF4, ATF3 might regulate expression of *HSPA5*, which encodes for a heat shock protein bound to GPX4 and protecting the latter from degradation [10].

ATF3 repressed *SLC7A11* expression, but transactivated *CHAC1* and *ASNS* expression in response to erastin. These results were not unexpected as binding of ATF3 to DNA can result in either transcriptional repression or activation [53]. ATF3 can form homodimer, or bind to its family members as well as many other transcription factors (e.g., p53, Sp1, NF-κB) [15, 54, 55], leading to distinct transcriptional outcomes. While a prevailing model proposes that ATF3 recruits and/or stabilizes transcriptional repressors/corepressors (e.g., HDAC1) to repress promoters [56, 57], our results that mutating the ATF3-binding sites decreased the activity of *SLC7A11* promoter (Fig. 4k) suggest that ATF3 more likely repressed *SLC7A11* expression through competing with transcriptional activators/coactivators for promoter binding. The BS-1/BS-2 sequence matches the consensus sequence of the C/EBP-ATF response elements, and thus these sites could be bound by both the C/EBP and the ATF/CREB family of transcription factors [58, 59], many of which are transcriptional activators. BS-1 and BS-2 sites are adjacent, separated by only 9 bp, and in different orientation (Fig. 4c). This unique feature might facilitate homodimerization of DNA-bound ATF3 and stabilize the ATF3-DNA interaction, thereby shielding these sites from access to transcriptional activators. Intriguingly, in addition to the –20 site, ATF3 appears to bind to its own promoter at another site proximal to the transcription start site [14]. Thus, ATF3 might repress its own promoter [32] with a mechanism similar to that for *SLC7A11*. In contrast, neither the *CHAC1* nor the *ASNS* promoter contains two adjacent ATF3-binding sites for promoting ATF3 to bind to DNA.

Another important finding from this study was that ATF3 repressed *SLC7A11* expression and promote ferroptosis independent of p53. While p53 can induce ATF3 expression [36, 37], ATF3 binds to p53 and stabilizes the tumor suppressor in response to DNA damage [15, 26, 60]. Although ATF3 was shown to reduce the p53 mRNA level in umbilical vein endothelial cells and keratinocytes [61, 62], we did not find that ATF3 expression decreased the p53 level in all of the cells that we tested. Intriguingly, while erastin slightly increased the p53 level (Fig. 1a, b), ATF3 appeared to be required for this induction (Fig. 1b). As the predicted p53-binding site (p53BS) in the human *SLC7A11* promoter was dispensable for p53-mediated repression of *SLC7A11* expression (Fig. 5b), our results raise an important question as to whether p53 represses *SLC7A11* promoter through interacting with ATF3. It is worth noting that p53 appears to play opposite roles in the regulation of ferroptosis. On one hand, p53 can promote oxidative stress-induced ferroptosis by repressing *SLC7A11* while activating *SAT1* expression [8, 63]. On the other hand, p53 suppresses erastin-induced ferroptosis by inhibiting DPP4 in colorectal cancer (CRC) cells or inducing p21 expression in non-CRC cells [64, 65]. However, p53-mediated suppression of ferroptosis requires preactivation of p53 [65]. As ATF3 could directly bind to the *SLC7A11* promoter and since *SLC7A11* overexpression could abolish ATF3-mediated ferroptosis, we concluded that ATF3-mediated repression of *SLC7A11* expression and suppression of system Xc^−^ might be the major mechanism by which ATF3 promotes ferroptosis induced by metabolic stress.

## Supplementary information

Supplemetal Figures
