# Supplementary material for: ATF3 promotes erastin-induced ferroptosis by suppressing system Xc–
Source: Cell Death Differ. 2019 Jul 4;27(2):662–75. doi: 10.1038/s41418-019-0380-z (PMC7206049; doi:10.1038/s41418-019-0380-z)
Supplement: Supplementary file 1 — Supplemetal Figures [file 41418_2019_380_MOESM1_ESM.pdf]

Supplementary Figures

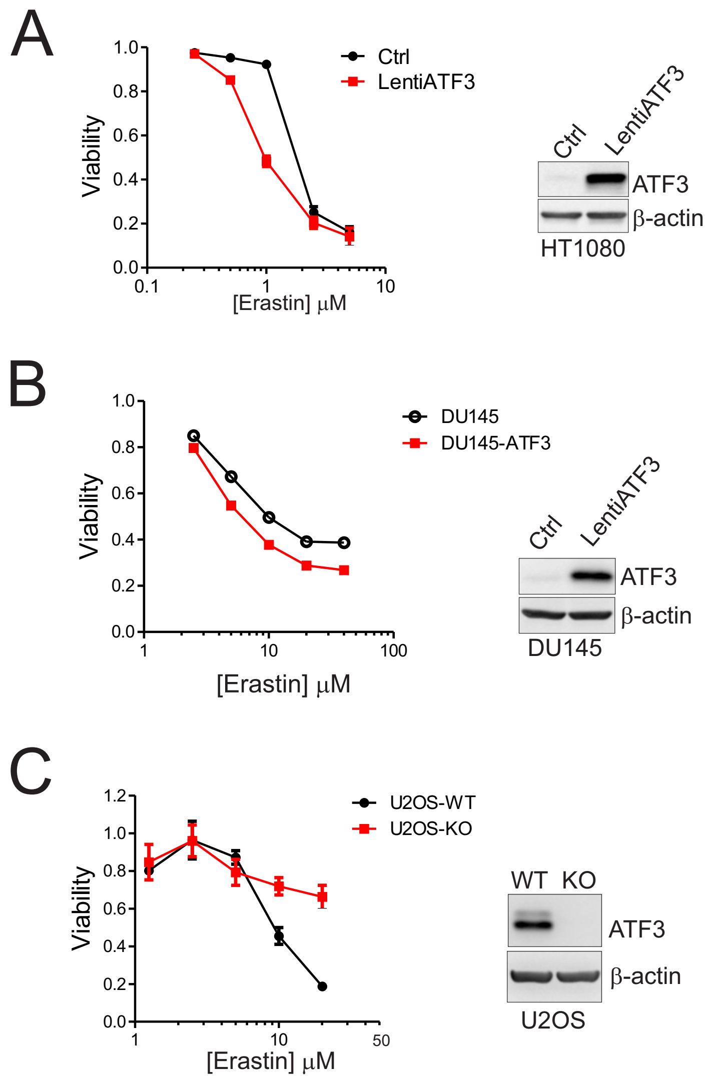

Fig S1. **ATF3 promotes erastin-induced ferroptosis in various cells.**
(A, B) HT1080 cells (A) and DU145 cells (B) infected with LentiATF3 or control viruses were treated with erastin for 24 h, and viability was determined by MTT assays. (C) *ATF3* expression was knocked out in UOS cells by CRISPR/Cas9. Cell viability after erastin treatments (24 h) was determined by MTT assays. The data are presented as mean ± SD.

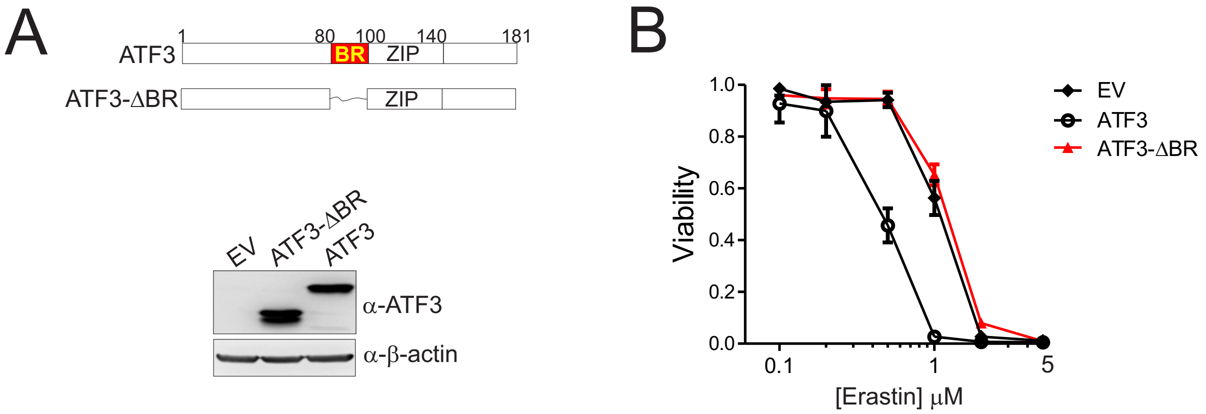

Fig S2. **ATF3-ΔBR overpression does not promote erastin-induced ferroptosis.**
HT1080 cells were transfected with ATF3-ΔBR, and a random clone expressing ATF3-ΔBR at a level comparable to that of HT1080-ATF3 cells was expanded for Western blotting (A), and then treated with erastin for viability assays (B). BR, basic region. ZIP, leucine zipper. The viability data are presented as mean ± SD.

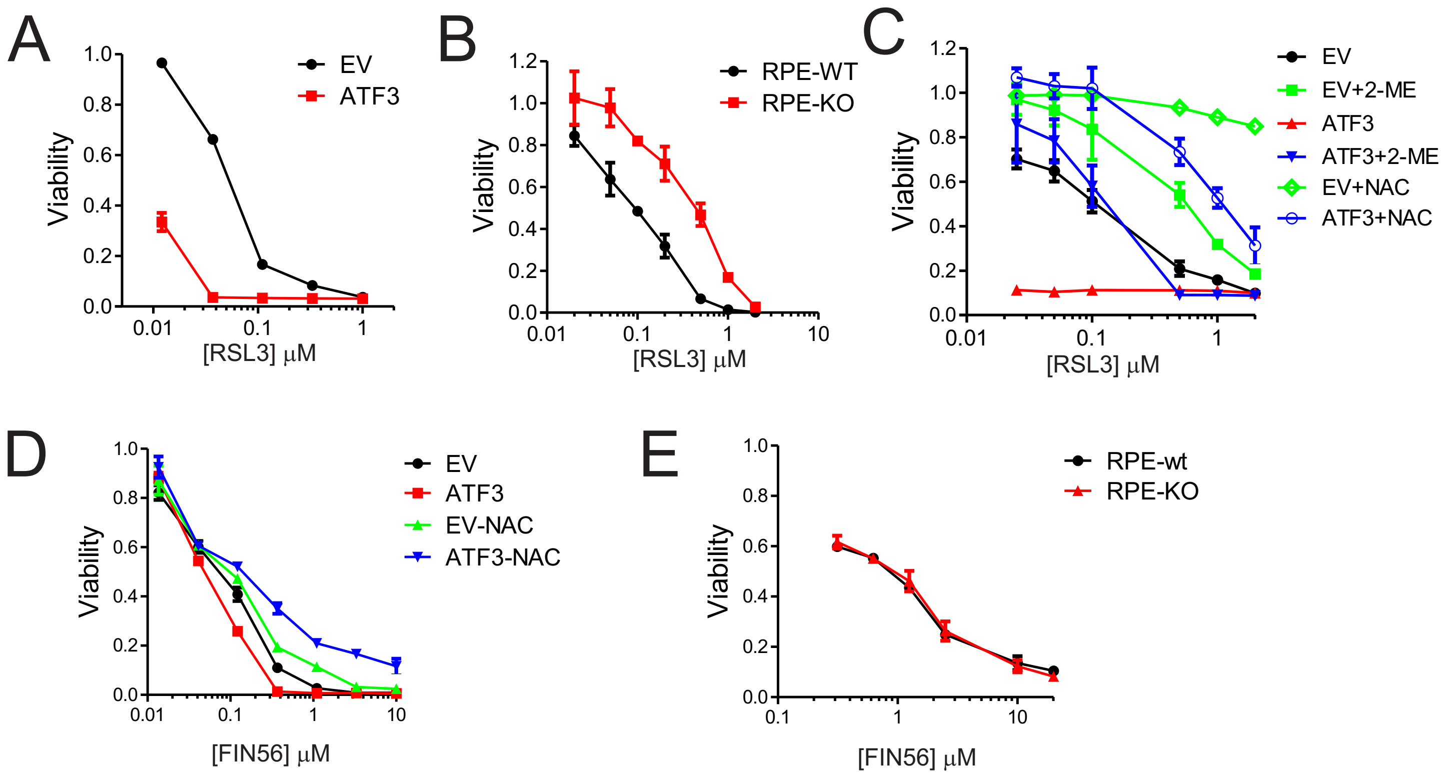

Fig S3. **ATF3 sensitizes cells to RSL3-induced, but not FIN56-induced, ferroptosis.**
(**A, B**) Indicated HT1080 and RPE cells were treated to RSL3 for 24 h and then subjected to viability assays. (**C**) Indicated HT1080 cells were treated with RSL3 with or without 2-ME (20 μM) or NAC (3mM) for 24 h, and subjected to MTT assays. (**D**) Indicated HT1080 cells were treated with FIN56 in the presence/absence of 3 mM NAC for 24 h, and subjected to MTT assays to measure cell viability. (**E**) Indicated RPE cells were treated with FIN56 for 24 h for MTT assays. The viability data are presented as mean ± SD.

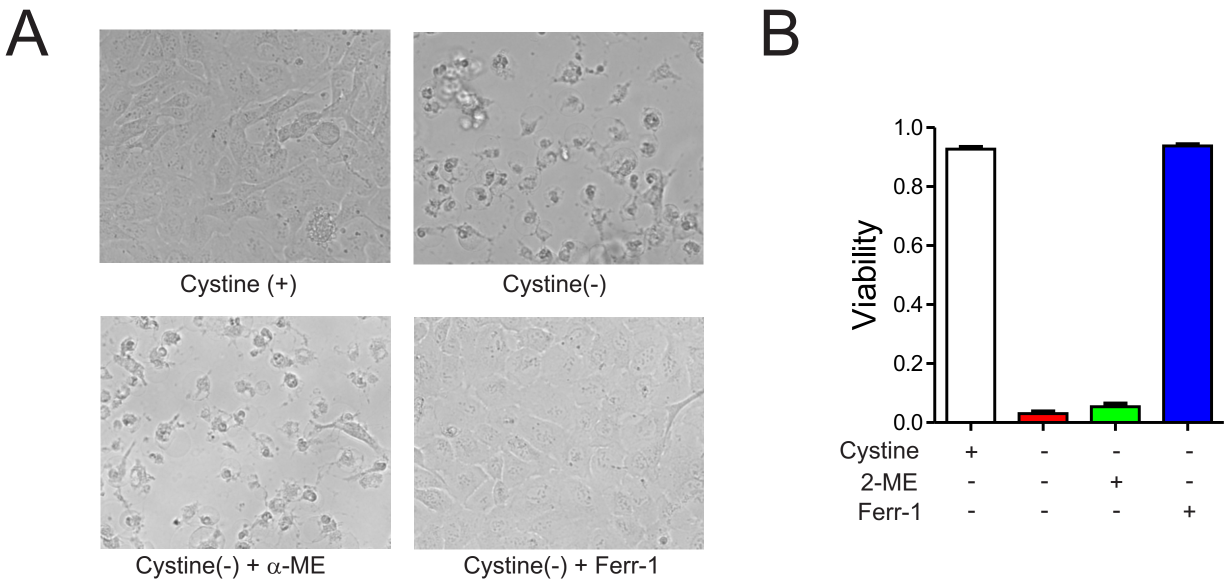

Fig S4. **2-ME does not rescue ferroptosis induced by cystine deprivation.**
(**A**) HT1080 were cultured in cystine-free DMEM (cystine(-)) in the absence/presence of 200 μM of 2-ME or 2 μM of Ferr-1 for 24 h, and their morphology was observed under a microscope. Note that the cells cultured in cystine-free medium show typical ferroptosis morphology. (**B**) Viable cells were counted after stained with Trypan Blue. Ferr-1 could rescue cell death induced by cystine deprivation, demonstrating that the type of cell death was ferroptosis. In contrast, 2-ME failed to rescue ferroptosis induced by cystine deprivation, suggesting that the general/nonspecific reducing and anti-oxidant activity of α-ME might not contribute to its rescuing effect on erastin-induced ferroptosis.

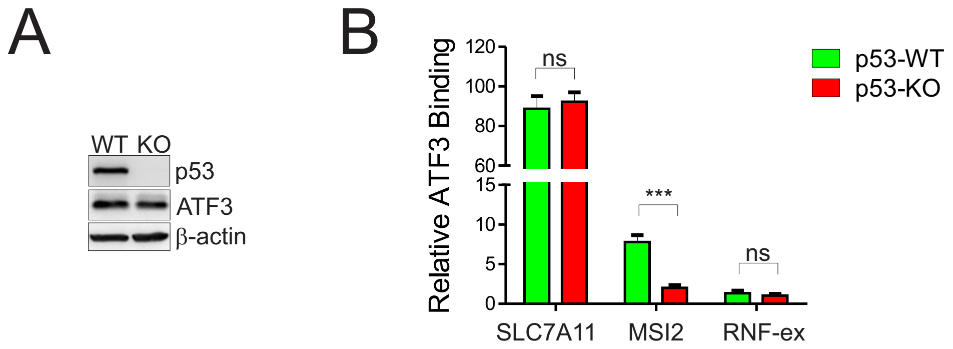

Fig S5. **ATF3 binds to the *SLC7A11* promoter in p53-knockout cells.**
(**A**) p53-wildtype (WT) and knockout (KO) HCT116 cells were treated with 2 μM of camptothecin (CPT) for 5 h to induce p53 activation, and then subjected to Western blotting to confirm that p53 expression was knocked out in the p53-knockout cells. (**B**) p53-WT and –KO HCT116 cells treated with CPT were subjected to chromatin immunoprecipitation with the ATF3 antibody. Relative ATF3 binding or enrichment was calculated as fold difference between the amount of DNA precipitated by the ATF3 antibody and the amount non-specifically precipitated by IgG. The data are presented as mean ± SD. ns, not significant; ***, $p<0.001$; Student's t-test.
